# The patient journey in Chronic Obstructive Pulmonary Disease (COPD): a human factors qualitative international study to understand the needs of people living with COPD

**DOI:** 10.1186/s12890-023-02796-8

**Published:** 2023-12-13

**Authors:** Nicola Scichilone, Andrew Whittamore, Chris White, Elena Nudo, Massimo Savella, Marta Lombardini

**Affiliations:** 1 Division of Respiratory Medicine, Department PROMISE, “Giaccone” University Hospital, University of Palermo, Palermo, Italy; 2 GP, Portsdown Group Practice, Portsmouth, UK; 3 Rebus Medical LTD, Bristol, UK; 4 Chiesi Farmaceutici S.P.A, Via Paradigna 131/A – 43122, Parma, Italy

**Keywords:** Chronic obstructive pulmonary disease, Assessment of healthcare needs, Qualitative research, Patient-centered care, Human-centered design, Human factors science, Pharmacologic therapy, Qualitative evaluation, Determination of healthcare needs, Multinational perspective, Quality of life, Quality of healthcare

## Abstract

**Background:**

Chronic obstructive pulmonary disease (COPD) is a common condition that causes irreversible airway obstruction. Fatigue and exertional dyspnoea, for example, have a detrimental impact on the patient’s daily life. Current research has revealed the need to empower the patient, which can result in not only educated and effective decision-making, but also a considerable improvement in patient satisfaction and treatment compliance.

The current study aimed to investigate the perspectives and requirements of people living with COPD to possibly explore new ways to manage their disease.

**Methods:**

Adults with COPD from 8 European countries were interviewed by human factor experts to evaluate their disease journey through the gathering of information on the age, performance, length, and impact of diagnosis, symptoms progression, and family and friends' reactions. The assessment of present symptoms, services, and challenges was performed through a 90-min semi-structured interview. To identify possible unmet needs of participants, a generic thematic method was used to explore patterns, themes, linkages, and sequences within the data collected. Flow charts and diagrams were created to communicate the primary findings. Following analysis, the data was consolidated into cohesive insights and conversation themes relevant to determining the patient's unmet needs.

**Results:**

The 62, who voluntarily accepted to be interviewed, were patients (61% females, aged 32–70 years) with a COPD diagnosis for at least 6 months with stable symptoms of different severity. The main challenges expressed by the patients were the impact on their lifestyle, reduced physical activity, and issues with their mobility. About one-fourth had challenges with their symptoms or medication including difficulty in breathing. Beyond finding a cure for COPD was the primary goal for patients, their main needs were to receive adequate information on the disease and treatments, and to have adequate support to improve physical activity and mobility, helpful both for patients and their families.

**Conclusions:**

These results could aid in the creation of new ideas and concepts to improve our patient’s quality of life, encouraging a holistic approach to people living with COPD and reinforcing the commitment to understanding their needs.

## Introduction

Chronic obstructive pulmonary disease (COPD) is defined by irreversible airway obstruction linked with comorbidities or systemic effects  [1]. COPD is a worldwide epidemic that contributes significantly to healthcare expenses due to high morbidity and mortality rates [2, 3]. The clinical assessment of fixed airflow limitation and symptoms such as coughing and wheezing determine a COPD diagnosis; nevertheless, COPD symptoms negatively impact the patient's daily activities and lifestyle [4]. Patients may encounter a variety of debilitating physical symptoms, resulting in functional loss and high degrees of psychosocial anguish [5–7].

Integrated approaches to disease assessment and management are required to better understand and address the burden of COPD symptoms from a patient's perspective [8].

According to a recent observational study, regardless of disease severity, more than half of COPD patients experienced symptoms during the whole 24-h day, and almost 80% of patients reported experiencing symptoms at least twice a day. Symptoms are linked to poor health, depression, anxiety, and poor sleep quality [9, 10].

Patients with COPD and comorbidities remain particularly challenging to manage because in Europe there is, generally, no guidance at the national level except in the UK, Slovenia, and Germany [11–13]. In Nordic countries and France, the management of patients with COPD is mainly performed by general practitioners with an inadequate level of assistance [14–16]. In other countries, patient management is performed at the discretion of the local structures, and the need for a comprehensive, holistic approach is looked forward [17–20]. Other chronic conditions increase symptom load, impair functional performance, and negatively impact health status; thus, management strategies must be adjusted accordingly [10].

Care plans, within the healthcare system, emphasize the importance of addressing these patients' particular physical, psychological, social, and spiritual needs through holistic supportive input offered as person-centered care [21]. Understanding the patient's perspective on their support requirements (those areas of living with COPD for which they require assistance, such as help controlling symptoms or accessing financial benefits) is critical to facilitating this approach. A recent systematic literature review has identified a whole range of support needs for COPD patients, based on the perspectives of the patients themselves [7].

Our human factor study aims to explore how COPD has affected the patients’ daily lives and the lives of those around them, through the assessment of symptoms, treatment, and service availability, identifying what challenges the patient faces in living with COPD, and which are the unmet needs in the different stages of the journey of care.

## Methods

This human factors COPD patient needs study was conducted in November 2022 by an ISO 13485 certified specialist human factors consultant (Rebus Medical Ltd), both in-person or remotely, via video call using the Zoom platform. Remote interviews were needed to enable more severe patients to attend the sessions and to ensure that the intended study sample was achieved. As for other qualitative analyses, a minimum of 48 participants were planned to be interviewed.

**Fig. 1:**
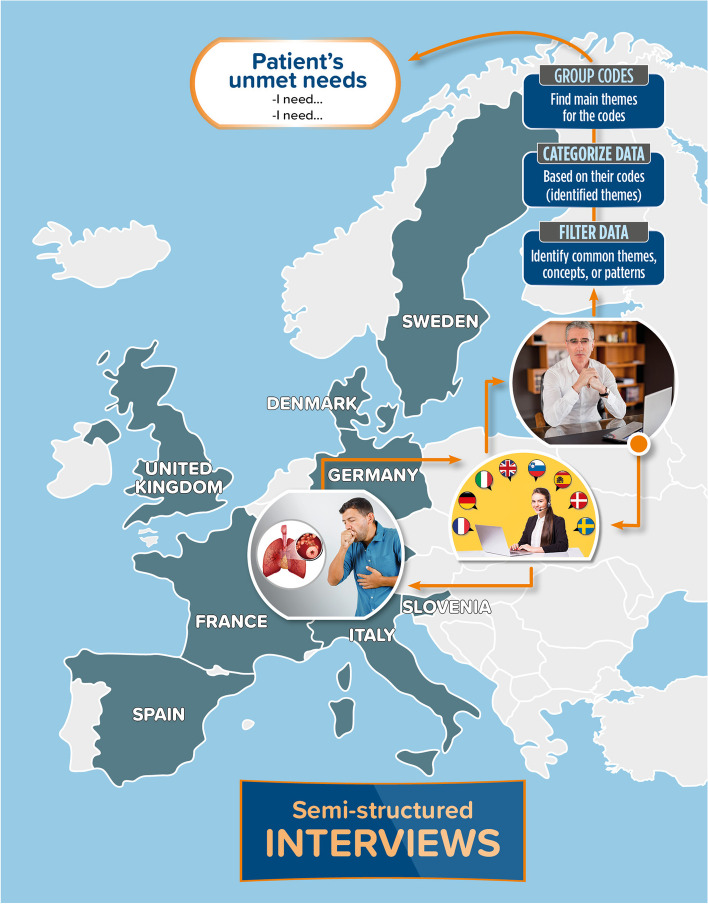
Summary of the study methods. Countries involved in the study are indicated in grey

Interviews were conducted on a 1–1 basis, with patients who voluntarily accepted to be interviewed from 8 countries: Denmark, France, Germany, Italy, Slovenia, Spain, Sweden, and the UK. Each interview was 90 min long and followed a semi-structured approach allowing for unscripted discussion when the participants’ responses raised new questions. For interviews that took place outside of the UK, a native-speaking moderator conducted the interview, whilst an interpreter translated the conversation live to a data analyst (Fig. 1).

Participants included in the study, aged 18 years or older, with a current COPD diagnosis, were screened for COPD severity according to GOLD criteria-2020-document [22] and voluntarily provided their informed consent.

Because the objectives were connected to identifying unmet requirements through video conference, the formative interviews were deemed low to minimal risk to participants and, thus, no formal approval to an Ethical Committee was required.

For interviews conducted in a language other than English, a simultaneous translator was recruited to enable a member of Rebus Medical staff to watch the interview listen to the translation, and record notes. Digital video recordings were collected to accurately account for each test session. Notes were verified at the end of each interview, while participant faces recorded on the videos were blurred to anonymize the footage. When all interviews were complete, the raw notes from each interview were collated and verified using the recorded videos in a master data capture spreadsheet.

The interviews were conducted to evaluate the journey of care through the collection of information on the gender, age, performance, length, and impact of diagnosis, symptoms progression, and family and friends’ reactions through questions that were designed on purpose to identify the unmet need and main challenges of each step of the patient’s journey. The evaluation of the current symptoms (fluctuations, flare-ups, alleviations, effect on sleep and daily activities including the use of electronic devices), services (health care providers support, insurance, available information on COPD), and challenges (in lifestyle, daily activities, treatments, symptoms management, emotional and environmental) was included in the semi-structured interview (Table 1).

**Table 1:** Overview of session flow

STAGE	DESCRIPTION	TIME
Interview: General questions	INTRODUCTION QUESTIONS An introduction to the study premise and exploration of the participant's background	5 min
Interview: Section A	DIAGNOSIS An exploration of the participant's experience during their diagnosis journey	10 min
Interview: Section B	CURRENT SYMPTOMS An exploration of what led participants to need treatment as well as how much symptoms of COPD impact patients’ lives and if these impacts vary throughout the day	5 min
Interview: Section C	CURRENT TREATMENTS A discussion with participants regarding their current treatment regime likes and dislikes of the current regime, how they manage adherence to their medication, and what support they receive	10 min
Interview: Section D	CURRENT SERVICES A focus on the additional services currently received by the participant in support of their COPD and how much contact they have with their health care provider	10 min
Interview: Section E	POSSIBLE CHALLENGES An exploration of the challenges and emotional feelings experienced by the participant, in order to understand the impact their condition has on their lifestyle	15 min
Close	Session close	5 min

As this was an exploratory insight interview, protocol deviations like alterations to the interviewer’s script to reformulate questions, ad hoc addition of questions and probes to the interviewer’s script to focus on points of interest specific to each participant, and changes to the interviewer’s script as the study progresses to allow for study learnings were permitted and expected.

A generic thematic approach was employed to uncover patterns, themes, links, and sequences within the data collected to identify probable unmet needs of participants through the patient journey of people living with COPD.

To communicate the major findings, flow charts, and diagrams were constructed. Following analysis, the data were synthesized and refined into cohesive insights and discussion themes pertinent to identifying the patient's unmet needs along the different stages of the patient journey.

## Results

A total of 62 patients (38—61% females) with COPD aged between 32 and 70 years (*N* = 1 aged 25–40 years, *N* = 42 aged 41–65 years, *N* = 19 aged > 65 years) were interviewed. Most of the patients (35—56%) had severe COPD (Table 2).

**Table 2:** Participant demographics by country and disease severity

		**UK**	**Italy**	**Germany**	**France**	**Denmark**	**Sweden**	**Slovenia**	**Spain**	**Total**
COPD	COPD patients interviewed per country	9	11	9	10	6	2	6	9	62
	Severe COPD patients per country	4	7	3	8	5	1	3	4	35
	Mild/moderate COPD patients per country	5	4	6	2	1	1	3	5	27

**Fig. 2:**
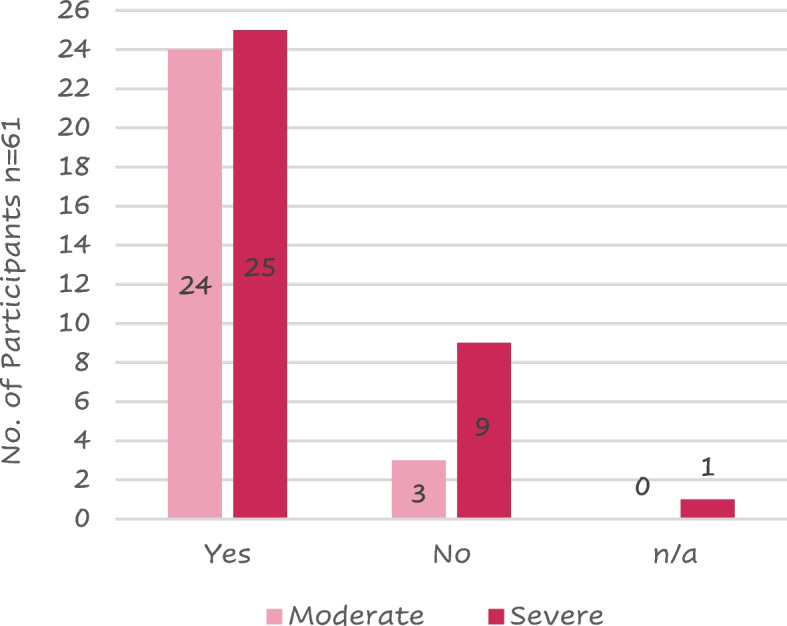
Distribution of patients that have ever been a smoker against COPD severity Legend: n/a = not available

Current- or past smokers were 49 (80%) of the 61 respondents. A larger proportion of patients with severe COPD (9/35, 26%) had never smoked compared to the moderate COPD patient group (3/27, 11%); in fact, 26 (74%) severe patients and 24 (89%) moderate were smokers or had smoked in the past (Fig. 2).

### Patient journey

#### Diagnosis

**Fig. 3:**
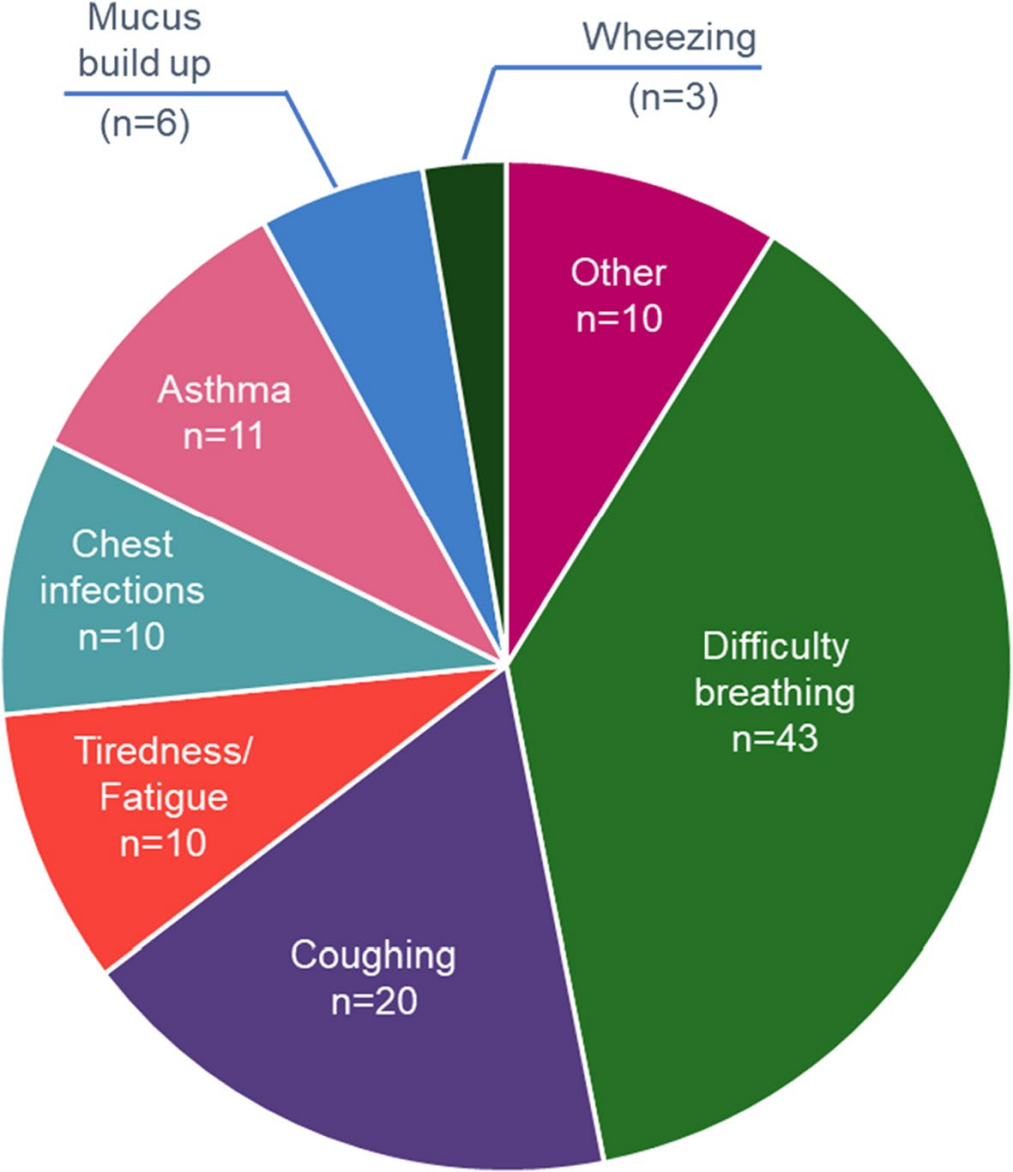
Patient’s reported signs and symptoms leading to COPD diagnosis Note—Other includes chest tightness, hereditary respiratory issues, persistent flare ups, unable to walk upstairs, difficulty talking, difficulty walking, difficulty swallowing, bronchitis as a child and headaches

A total of 113 symptoms of COPD were recorded because most patients reported more than 1 symptom at the onset of the disease; 78 (69%) of these symptoms were related to dyspnoea. The highest reported symptoms were difficulty breathing and coughing (Fig. 3).

Fourteen (30%) of the 46 respondents referred to being diagnosed with COPD more than 1 year after initial symptoms, while 6 (13%) were diagnosed from 7 to 12 months from the onset of symptoms. Ten (64%) of the 14 requiring > 1 year for their diagnosis had severe COPD.

**Fig. 4:**
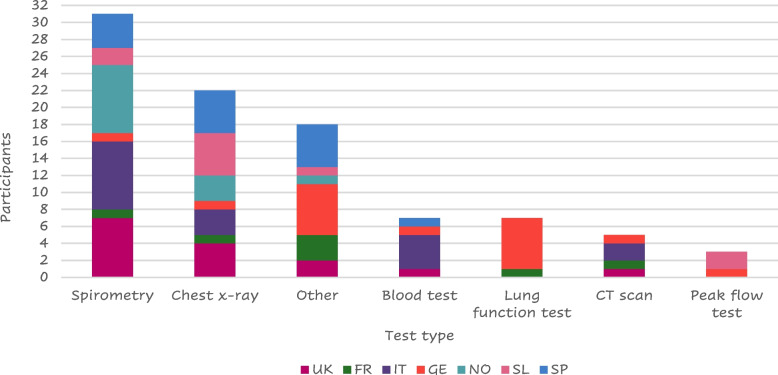
Tests performed at the visit of diagnosis Legend: FR = France, GE = Germany, IT = Italy, SL = Slovenia, SP = Spain, NO = Northern (Sweden Denmark), UK = United Kingdom. “Other” includes: MRI, pressure cabin test, swabs collected, endoscope to check lungs, chamber, PET scan, Blood taken from the ear, blood gas test, oxygen saturation, walking/ running tests, echocardiogram, pulse oximeter/O_2_ saturation, sleep test

Most of the 56 patients who answered (41 – 73%) were diagnosed by a lung specialist mainly using spirometry (Fig. 4).

**Fig. 5:**
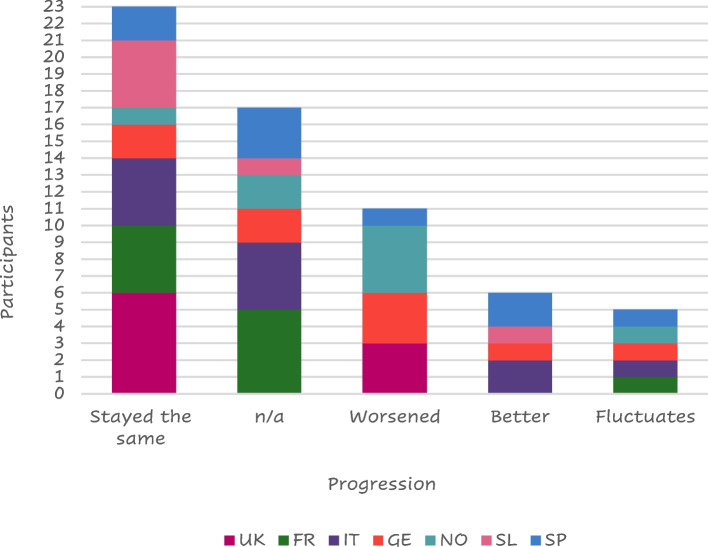
Symptom progression Legend: FR = France, GE = Germany, IT = Italy, SL = Slovenia, SP = Spain, NO = Northern (Sweden Denmark), UK = United Kingdom, n/a = not available

About half of the responders (23 of 45 – 51%) felt their symptoms stable from the diagnosis (Fig. 5).

Thirteen (29%) of those interviewed stated that their family and friends were supportive at the time of COPD diagnosis while 8 (18%) were worried about the diagnosis. Seven of them received no reaction from their family or friends and a further 7 did not tell anyone about their diagnosis. ‘Other’ reactions that were received from family and friends included: acceptance, anger, fear, shock, anguish, and expected, while some patients “prefer not to speak about it”.

The COPD diagnosis hurt 26 (58%) of the responders who described a negative impact of their COPD diagnosis, mainly because of their inability to be active, while 13 of them (29%) felt a positive impact mainly because they stopped or reduced smoking (Table 3).

**Table 3:** Ways in which patients' lives have been impacted by COPD since diagnosis

Positive impacts	Negative impacts
Given up/ reduced smoking (*n* = 6)	Social life (*n* = 5)
	Hobbies (*n* = 3)
**‘others’** include; Awareness and started taking care of my life	**‘others’** include; accessibility, motivation, energy levels, can’t have a normal life

Six (19%) of the 31 patients who provided details on the reason for quitting smoking reported they received more information about how to give up smoking and the risks associated with smoking, 3 patients mentioned some form of medication to support smoking cessation may have helped them give up, and 2 patients reported that they would give up for a family member but would struggle to have the motivation to do it themselves. Three patients reported that nothing would have helped them stop smoking while 8 patients reported that, despite knowing the impact smoking has, they still chose to smoke. Other suggestions to stop smoking reported by participants included: the threat of death, vaping if the smoking affected their fitness, cigarettes stopped being sold, stopping because of asthma and its diagnosis, quitting when they were in the hospital for a week giving it up after then, or because the smell was horrible.

### Symptoms

A total of 59 patients answered about their changes in symptoms throughout the day; seventeen (29%) felt no changes while 13 (22%) worsened in the morning, 11 (19%) worsened at night, and 6 (10%) worsened both in the morning and at night.

**Fig. 6:**
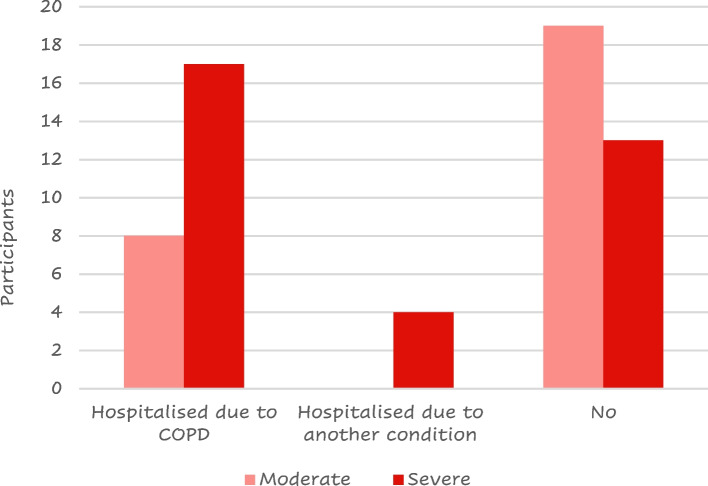
Number of patients that have experienced a COPD flare-up by COPD severity

Twenty-five (41%) of the 61 responders were hospitalized due to a COPD flare-up at least once after their COPD diagnosis; most of them had severe disease (Fig. 6).

Seven (30%) of the 23 patients who took any action to alleviate their symptoms, before seeing a doctor and getting a diagnosis, reduced their physical exercise to not trigger symptoms. While others were more vigilant with their health, received help from family and friends, or used inhalers, a rescue pack, or menthol sweets.

Thirty seven out of 58 participants reported sleep disruption. Of these, 12 (32%), reported disruption due to COPD while 10 (17%) had sleep negatively affected by another condition. Other causes for patients’ sleep disruption included coughing, the need to change sleeping positions, and cold weather.

Patients reported needing more support including more information about their condition, financial support for transportation, improved treatment options, accessibility badges, and help in carrying out chores in the house such as cooking, cleaning, and general housekeeping. Some patients also indicated a wish for personal training. Some patients were unaware of what type of support they may require or what type of support could be available to them while others were looking for a different inhaler or treatment to alleviate their cough or a device that assists deep breathing, transplant, a dog or a sport requiring a limited physical effort that would help them be more active, and/or meeting a COPD support group.

About half of the respondents (26/56 – 46%) used electronic devices to monitor their health status including a finger pulse oximeter (*n* = 9), smartwatch (*n* = 8), or a blood pressure cuff (*n* = 5).

### Treatments

A total of 64 responses were collected from the 58 patients who shared their opinion on the treatment they were utilizing; 33 (52%) of the feedback was positive (Table 4).

**Table 4:** Patients’ feeling about their current prescribed treatment/therapy regime

Positive (*n* = 33)	Negative/Other (*n* = 11)	Neutral (*n* = 20)
Working/Good Non-invasive Increased dose frequency Take when needed Administration is easy **Additional comments**; Concern about material waste	No therapeutic effect Patient is non-compliant Psychological constraints/ remembering to take medication is a burden Decreased dose frequency **Additional comments**; Throat infections, don’t like any treatments, but has no choice	Simple Prefers inhalers to tablets **Additional comments**; Concern about the hygiene of the mouthpiece

While 20 (31%) of the respondents felt neutral about their current prescribed treatment, 11 (17%) reported either that their medicine had "no therapeutic impact", that they faced "psychological restraint" with their prescribed regime, or that they had issues with treatment compliance.

Six (12%) of the 52 respondents confirmed using digital or analogic reminders to take their dose. Three patients were currently using a dose counter on their device to remind them if their doses had not been taken, and two patients were using a timer on their mobile phones to remind them when their next dose was due. One participant used digital/analogic support but did not indicate which.

The main strategies used to remind them to take their medication include:

- leaving the medication in a specific location to prompt them to take their dose at the correct time,
- relying on habit or routine to prompt them to take their medication,
- taking the COPD medication at the same time as other medications,
- feeling unwell to prompt themselves to take their medication.

A total of 32 (56%) of the 57 respondents reported missing a medicine dose; eight of them cited a change in their schedule or routine as its cause. Other reasons for missing a dose reported by patients included: not taking the medication seriously, forgetting to take their dose in the evening, forgetting to bring their medication with them when leaving the house, a change in their environment, a missed medication delivery, and “not taking regular doses”.

The primary reasons why patients appreciate their present treatments were the drug's functionality (*n* = 18), the device design (*n* = 10), the convenience of use (*n* = 8), and the medication's quick and uncomplicated administration (*n* = 5). Other patients expressed liking for current medication including feeling comfortable with their present treatment, feeling in charge, and independence.

On the other hand, the device design (*n* = 14), the necessity to take their medication (*n* = 8), and the side effects of the drug (*n* = 5) were the most reported characteristics that patients disliked therapy. Other reported reasons included uncertainty about what the treatment is supposed to do, a sense of guilt when their medication is forgotten, the fact that they are still limited in their activity, and the sensation or taste inside their mouth. Three patients stated that they did not enjoy their current prescribed treatment. "You have to accept what is available," one patient said. Other patients referred detest having to take their medications daily.

About two-thirds (*n* = 34 – 67%) of those polled (*n* = 51) claimed no involvement with the selection of their present treatment option.

Most of the patients (*n* = 42 – 69% of the 61 respondents) reported receiving training for the use of their current treatment. The remaining 31% of the patients did not receive any training, reporting that they “would have liked more formal training, the current device is more complex”, or believed it “could have been useful to receive training and would have loved the explanation, demo training”. Three patients also stated that they did not need training, whether they received it or not.

Twenty-two (52%) of the 42 patients that received training, thought that it was effective and only 5 (12%) did not believe their training to be effective. Fifteen (36%) of patients who received training did not provide feedback on the efficacy of the training they received.

**Fig. 7:**
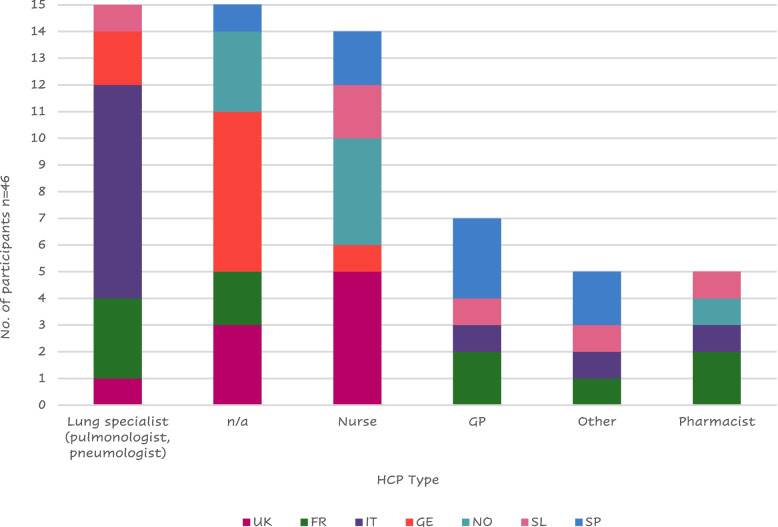
Health care provider (HCP) that administered training to patients by country Legend: FR = France, GE = Germany, GP = General Practitioner; IT = Italy, SL = Slovenia, SP = Spain, n/a = not applicable; NO = Northern (Sweden Denmark), UK = United Kingdom

Eight Italian patients reported receiving instruction mostly from a lung specialist, while the majority of British (*n* = 5) and Nordic (*n* = 4) patients reported receiving training primarily from a nurse (Fig. 7); this is probably due to the different structures of the national health systems.

One Italian patient stated he received no specific training but was told by his pneumologist to look inside the package and read the instructions; a Frenchman mentioned that his wife was a doctor, so she just showed him how to use the device. Other participants’ training was received at meetings of a lung association from the pharmacists or at a live course organized by the doctor or during rehabilitation.

Six (18% of the 34 respondents) received help from their family or friends to find training materials or treatment information. Most patients received help to find further information and one participant mentioned that he was able to speak to a relative with COPD.

Six (15%) of the 41 respondents had gone online for help with their equipment (looking for tutorials online on forums and finding animated videos on how to use their inhalers). The main reasons for not using the internet for support were a lack of trust in online information ("would rather trust a doctor than go online"), an unwillingness to read more about their condition due to a fear of "reading too much" and becoming "depressed" if they investigated their disease. Other patients did not feel the need for additional support from the internet because their devices were "easy to use" or they wouldn't need further support due to their disease. One patient stated that he looked online and "found it strange that the messages were exclusively for persons with moderate to severe COPD, with only a few messages from people with mild COPD".

### Services

**Fig. 8:**
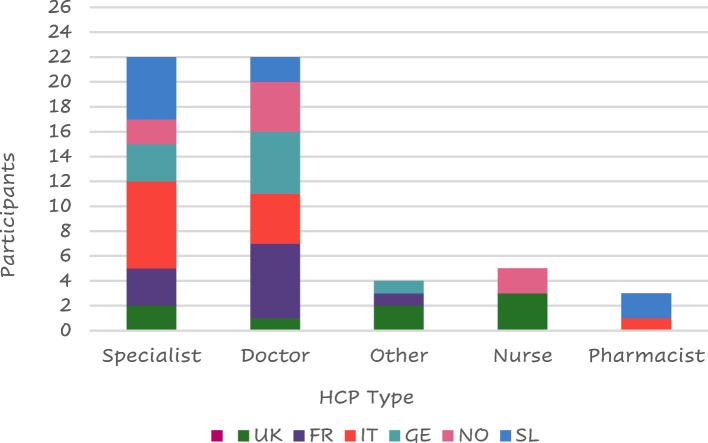
Type of HCP support by country Legend: FR = France, GE = Germany, IT = Italy, SL = Slovenia, SP = Spain, NO = Northern (Sweden Denmark), UK = United Kingdom

Lung specialists were the health care providers (HCPs) who most frequently provided support to patients with COPD (*n* = 24/60—40%) followed by general practitioners (23 – 38%) (Fig. 8); only 3 patients reported not having received any support.

**Fig. 9:**
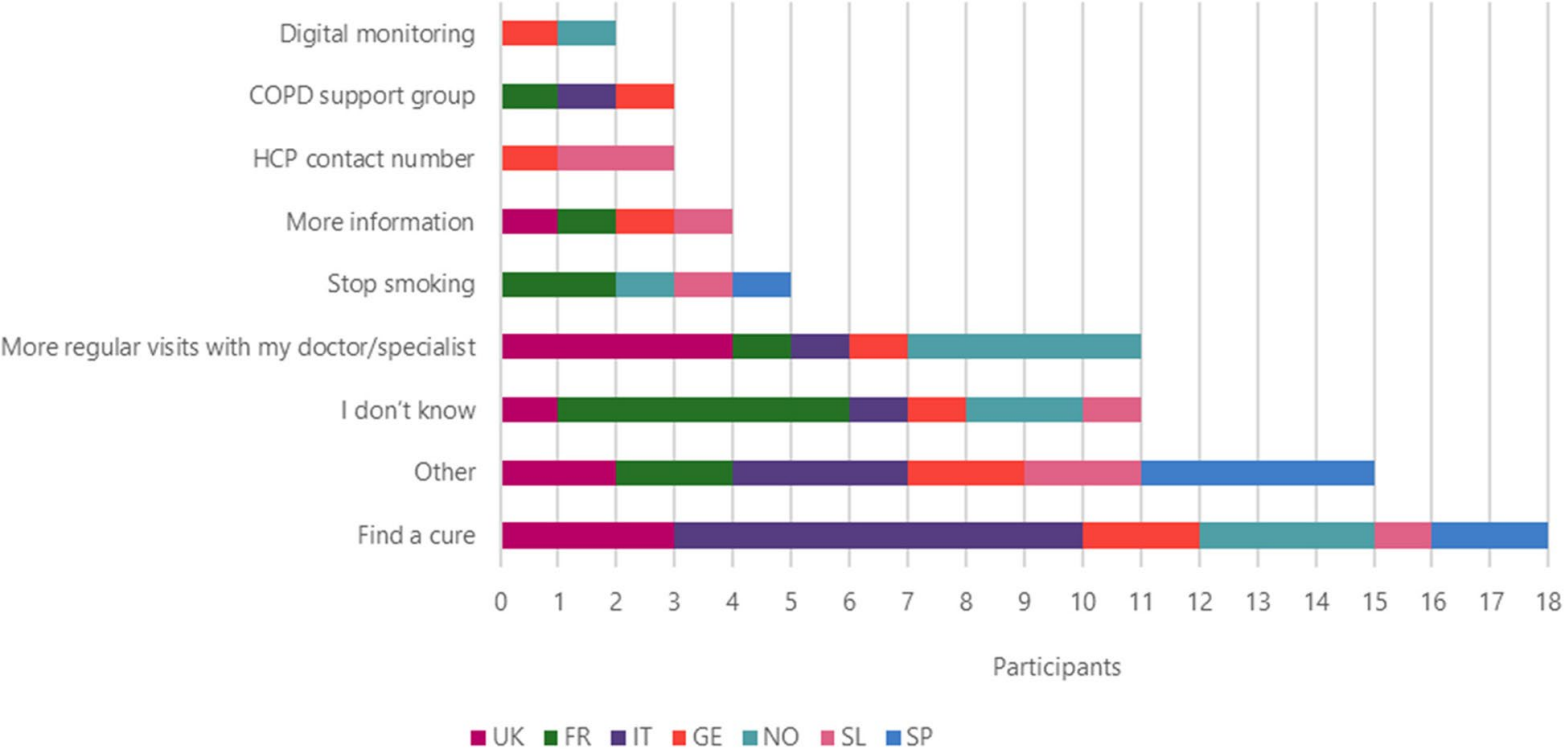
Improvements that patients wish to be made to improve their life by country Legend: FR = France, GE = Germany, IT = Italy, SL = Slovenia, SP = Spain, NO = Northern (Sweden Denmark), UK = United Kingdom

The most frequent answers to the question “If you had a magic wand what would you wish for to improve your life with COPD?” were to find a cure (*n* = 18), followed by more regular visits from their doctor/specialist (*n* = 11), stop smoking (*n* = 5), more information (*n* = 4), HCP contact number and COPD support group (*n* = 3), and digital monitoring (*n* = 2) (Fig. 9).

Other improvements that patients wish for include: access to new drugs, information about COPD, current and new drugs, reduced side effects, holding COPD workshops, investment in more research, provide cheaper treatment options, new lungs, something to help be more active, to be told that they would not need to take medication anymore, a new type of drug delivery that wouldn’t need to be taken with patient everywhere (like a nicotine patch), instant relief and doctors and nurses to be more humane.

**Fig. 10:**
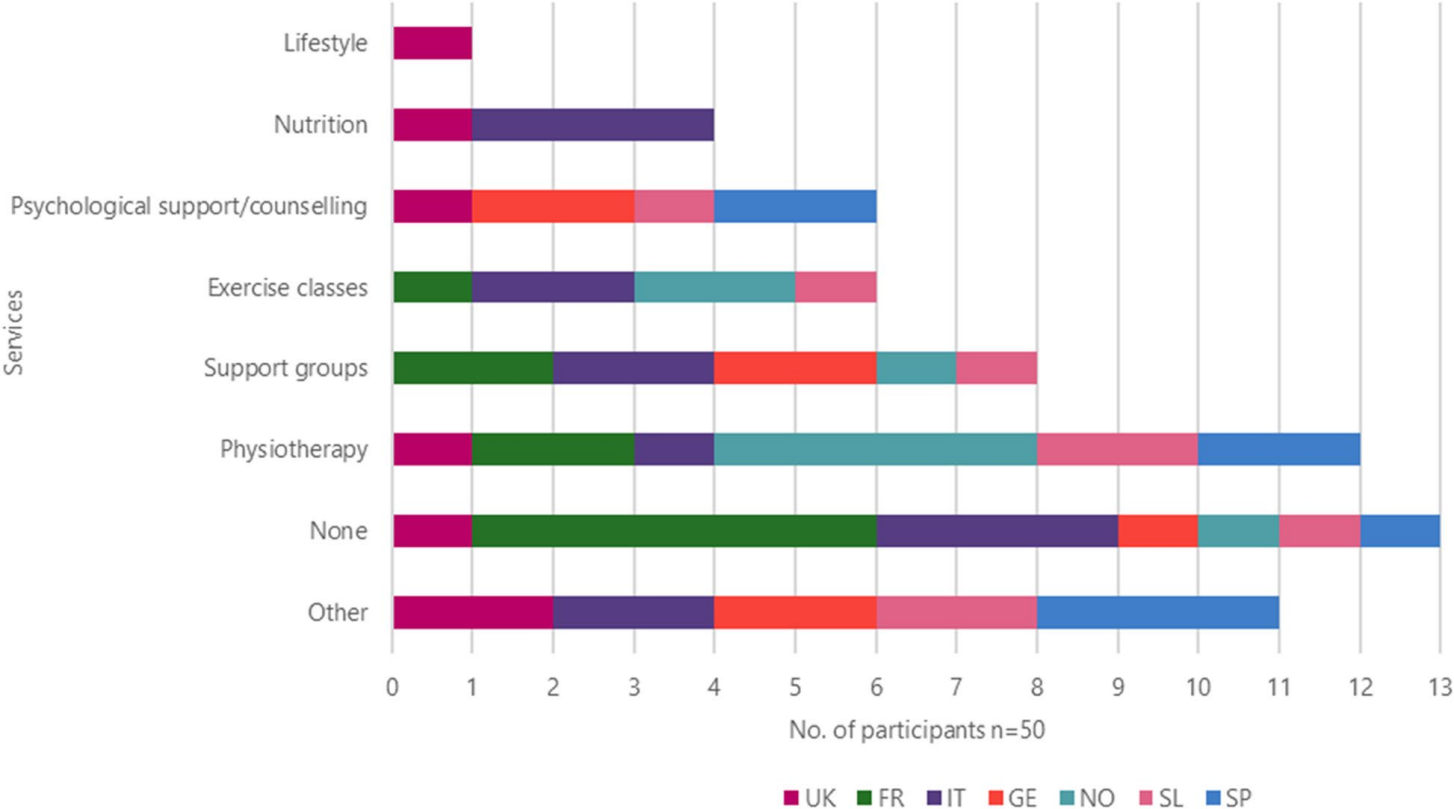
Other services the patient would like to use by country Legend: FR = France, GE = Germany, IT = Italy, SL = Slovenia, SP = Spain, NO = Northern (Sweden Denmark), UK = United Kingdom

Other services they felt were useful for them included physiotherapy (*n* = 12), the use of support groups (*n* = 8), exercise classes and psychological assistance (*n* = 6), nutrition (*n* = 4) while 1 patient from the UK suggested lifestyle (Fig. 10).

Other services that patients would like to use included easier access to their HCP, paid, private physiotherapy sessions, smoking cessation support, disability card, training (videos and tutorials) including emergencies, lung transplants, more information about new drugs and the benefits of medication, hear more from doctors and pharmacists, and workshops for families and friends to help them understand what patients are going through.

Even if 3 patients reported having insurance covering additional services, they were generally unaware of the support they could receive through medical insurance. Many had concerns that such services would cost more money.

### Challenges

**Fig. 11:**
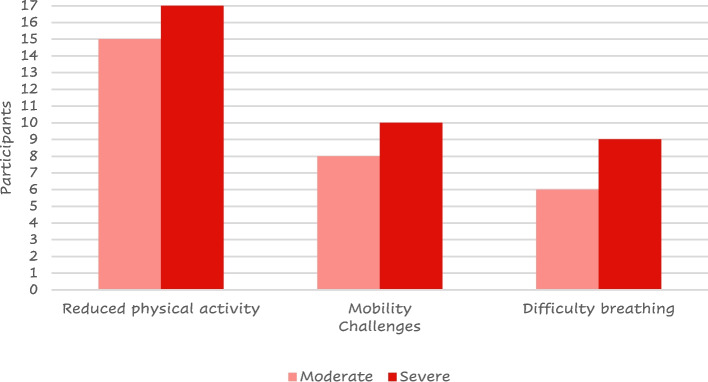
Most reported challenges by COPD severity

All the patients included in the study provided a total of 122 daily challenges they must face. 53 (43%) of the responses were related to their lifestyle. Reduced physical activity was referred by more than half (*n* = 32) of them and difficulty in mobility was reported by 16; 28 (23%) reported challenges with their symptoms or medication (mainly difficult breathing, *n* = 15) (Fig. 11) while 13 (11%) reported emotional challenges including anxiety, depression, embarrassment due to symptoms or treatment, fear of the conditioning worsening, people recognizing they have a condition, acceptance of the condition and dependence on the medication.

## Discussion

The objective of this human factors research was to identify the unmet needs along the different stages of people living with COPD through a one-to-one, semi-structured interview exploring the patient’s feelings and attitudes toward their journeys with the disease.

Differently from other studies exploring similar aspects of the impact of the disease on patient’s daily life where the data belong to medical databases, [4, 6, 7, 9, 10, 23] the current approach is unique, in that it systematically investigates the patient’s feelings in a structured fashion, thus allowing us to better understand the patient’s emotions, which is becoming a relevant aspect of COPD management [7, 24]. Furthermore, because of the consistent and wide heterogeneity between the different countries, patients included in this study could have been considered representative of the entire population of European patients with COPD.

The patient reported feelings highlighted that reduced physical activity, mobility challenges, and difficulty breathing resulted as the main challenges in daily life. According to the current international guidelines on COPD management, [22, 25, 26] physical activity is encouraged and monitored to evaluate the prognosis or looked forward to as a target for the evaluation of the treatment efficacy. [25] Our results confirm that patients perceive COPD as the cause of their reduced physical activity, [27] having a strong impact on their self-perception. Differently from other studies where increased physical activity was observed independently from patients’ counseling, [28] general psychological support and accepting their mobility challenges were described as important aims by the patients. Our patients felt reduced mobility as one of their main challenges; aids to improve mobility were described in the available literature as crucial to maintaining the patient’s independence [7] and have been included in the 2023 GOLD guidelines [29].

The HCP approach is mainly focused on improving the patient’s breathlessness and exercise intolerance [22, 25]; the feeling depicted by the interviewed patients confirms the lack of information about how to manage breathlessness. [30] The only positive aspect of the COPD diagnosis, reported by 6 of the interviewees, was smoke quitting. Patients frequently feel angry and depressed when they think about the difficulties they have described. Participants discussed a variety of coping mechanisms to deal with these difficulties, including cutting back on physical activity, making sure they stayed active (as much as possible), and utilizing their rescue inhaler as a preventative step.

About one-fourth of the patients did not report having performed spirometry at diagnosis; as spirometry is the landmark of diagnosis; any other method is not gold standard and subjected to criticism [22]. Because of the qualitative nature of this study, we cannot exclude that this issue was linked to the patient’s reduced memory at the time of diagnosis.

As observed in other studies, [31] negative behavior has a strong influence on the patient’s quality of life. Patients in the current study generally felt negative emotions before receiving their diagnosis; however, a supportive role of relatives and caregivers was referred by interviewed subjects at the time of diagnosis. About forty percent of patients complained of having waited long before the diagnosis. When asked about the impact of their current treatment, participants gave primarily positive feedback and commonly described their current therapy as “good” and doing its job. Even if most of the patients included in our study felt stable symptoms, some were still looking for a “miraculous” cure. The need for support beyond just pharmacological treatments, such as psychological support and physiotherapy, became clear through the in-depth discussions with patients, confirming the requirement for an integrated and patient-tailored interview to identify the profile of each patient [27, 32] to share the most appropriate interventions in the periodic visits, without the need of the patient’s hospitalizations to allow the introduction of new therapies suggested by other research [33].

As expected, our results show that the information about COPD and the training on both the disease and treatment were provided by different HCPs in various European countries. However, patients often felt that they were not provided with enough information at the point of diagnosis regarding the condition itself or the range of treatment options available. Some felt they did not receive adequate training on how to take their medication correctly, whereas others highlighted that the public should be made more aware of the condition, in general, to help them feel accepted and understood by their family and friends. When asked about the current support they were receiving for their disease, patients reported wanting more information about their clinical condition or treatment options, more regular visits with their HCP, smoking cessation assistance, and support in their day-to-day lives such as housework and improved accessibility, confirming the need of self-management education and skills training highlighted by other authors [22, 25, 26]. However, many patients were unsure or unaware of what support/services were available to them or did not feel they needed any additional support.

This study had a qualitative approach and was, thus, not designed to provide any definitive answer to a study hypothesis. Differently from other studies on general populations of patients with COPD where males and elderly are the most frequent patients [34, 35], those who agreed to participate in this study were mostly women and aged between 42 and 65 years. Due to the inclusion of patients that could not be fully representative of the global patients with COPD and the study approach, the outcomes have to be properly generalized. Furthermore, the nature of the study required interviews to be carried out in the participant’s local language with the use of translators to support analysis leading to a potential loss of nuance in meaning.

In conclusion, the current findings show that an apparent discrepancy exists between the traditional lung functional and pharmacological approaches in diagnosing and managing COPD and patient’s needs and challenges in daily activities. In this respect, human factor studies play a relevant role in intercepting gaps in the care of people suffering from COPD, encouraging a novel holistic approach when designing clinical research or shepherding patients along their COPD daily journey.

## Data Availability

The data that support the findings of this study are available from Chris White (Rebus Medical), but restrictions apply to the availability of these data, which were used under license for the current study, and so are not publicly available. Data are not available without permission of Chiesi Farmaceutici.

## References

[1] Decramer M, Janssens W, Miravitlles M. Chronic obstructive pulmonary disease. Lancet. 2012 [cited 2023 May 29];379(9823):1341–51. Available from: https://pubmed.ncbi.nlm.nih.gov/22314182/. 10.1016/S0140-6736(11)60968-9 PMC7172377 22314182

[2] Roggeri A, Micheletto C, Roggeri DP. Outcomes and costs of treating chronic obstructive pulmonary disease with inhaled fixed combinations: the Italian perspective of the PATHOS study. Int J Chron Obstruct Pulmon Dis. 2014 Jun 5 [cited 2023 May 29];9:569–76. Available from: https://pubmed.ncbi.nlm.nih.gov/24940053/. 10.2147/COPD.S65693 PMC4051514 24940053

[3] Blasi F, Cesana G, Conti S, Chiodini V, Aliberti S, Fornari C, et al. The clinical and economic impact of exacerbations of chronic obstructive pulmonary disease: a cohort of hospitalized patients. PLoS One. 2014 Jun 27 [cited 2023 May 29];9(6). Available from: https://pubmed.ncbi.nlm.nih.gov/24971791/. 10.1371/journal.pone.0101228 PMC4074190 24971791

[4] Rennard S, Decramer M, Calverley PMA, Pride NB, Soriano JB, Vermeire PA, et al. Impact of COPD in North America and Europe in 2000: subjects’ perspective of Confronting COPD International Survey. Eur Respir J. 2002 Oct 1 [cited 2023 May 29];20(4):799–805. Available from: https://pubmed.ncbi.nlm.nih.gov/12412667/. 10.1183/09031936.02.03242002 12412667

[5] Sundh J, Ekström M. Persistent disabling breathlessness in chronic obstructive pulmonary disease. Int J Chron Obstruct Pulmon Dis. 2016 Nov 9 [cited 2023 May 29];11(1):2805–12. Available from: https://pubmed.ncbi.nlm.nih.gov/27877034/. 10.2147/COPD.S119992 PMC5108478 27877034

[6] Ouellette DR, Lavoie K. Recognition, diagnosis, and treatment of cognitive and psychiatric disorders in patients with COPD. Int J Chron Obstruct Pulmon Dis. 2017 Feb [cited 2023 May 29];12:639–50. Available from: https://pubmed.ncbi.nlm.nih.gov/28243081/. 10.2147/COPD.S123994 PMC5317263 28243081

[7] Gardener AC, Ewing G, Kuhn I, Farquhar M. Support needs of patients with COPD: a systematic literature search and narrative review. Int J Chron Obstruct Pulmon Dis. 2018 Mar 26 [cited 2023 May 29];13:1021–35. Available from: https://pubmed.ncbi.nlm.nih.gov/29628760/. 10.2147/COPD.S155622 PMC5877489 29628760

[8] Spruit MA, Singh SJ, Garvey C, Zu Wallack R, Nici L, Rochester C, et al. An official American Thoracic Society/European Respiratory Society statement: key concepts and advances in pulmonary rehabilitation. Am J Respir Crit Care Med. 2013 Oct 15 [cited 2023 May 29];188(8). Available from: https://pubmed.ncbi.nlm.nih.gov/24127811/. 10.1164/rccm.201309-1634ST 24127811

[9] Miravitlles M, Worth H, Soler Cataluña JJ, Price D, De Benedetto F, Roche N, et al. Observational study to characterise 24-hour COPD symptoms and their relationship with patient-reported outcomes: results from the ASSESS study. Respir Res. 2014 Oct 21 [cited 2023 May 29];15(1). Available from: https://pubmed.ncbi.nlm.nih.gov/25331383/. 10.1186/s12931-014-0122-1 PMC4220061 25331383

[10] Jones PW, Watz H, Wouters FM, Cazzola M, COPD: the patient perspective.  (2016). cited 2023 May 29. Available from:.

[11] Škrgat S, Triller N, Košnik M, Susič TP, Petek D, Jamšek VV (2017). Priporočila za obravnavo bolnika s kronično obstruktivno pljučno boleznijo na primarni in specialistični pulmološki ravni v Sloveniji. Zdravniski Vestnik.

[12] Mehring M, Donnachie E, Fexer J, Hofmann F, Schneider A. Disease management programs for patients with COPD in Germany: a longitudinal evaluation of routinely collected patient records. Respir Care. 2014 [cited 2023 Nov 15];59(7):1123–32. Available from: https://pubmed.ncbi.nlm.nih.gov/24222706/. 10.4187/respcare.02748 24222706

[13] Overview | Chronic obstructive pulmonary disease in over 16s: diagnosis and management | Guidance | NICE. [cited 2023 Nov 15]. Available from: https://www.nice.org.uk/guidance/ng115.

[14] Molin KR, Søndergaard J, Lange P, Egerod I, Langberg H, Lykkegaard J. Danish general practitioners’ management of patients with COPD: a nationwide survey. Scand J Prim Health Care. 2020 [cited 2023 Nov 15];38(4):391–8. Available from: https://pubmed.ncbi.nlm.nih.gov/33164618/. 10.1080/02813432.2020.1842964 PMC7782274 33164618

[15] Sandelowsky H, Natalishvili N, Krakau I, Modin S, Ställberg B, Nager A. COPD management by Swedish general practitioners – baseline results of the PRIMAIR study. Scand J Prim Health Care. 2018 Jan 2 [cited 2023 Nov 15];36(1):5. Available from: /pmc/articles/PMC5901441/. 10.1080/02813432.2018.1426148 PMC5901441 29334861

[16] Meeraus W, Wood R, Jakubanis R, Holbrook T, Bizouard G, Despres J, et al. COPD treatment pathways in France: a retrospective analysis of electronic medical record data from general practitioners. Int J Chron Obstruct Pulmon Dis. 2018 Dec 18 [cited 2023 Nov 15];14:51–63. Available from: https://www.dovepress.com/copd-treatment-pathways-in-france-a-retrospective-analysis-of-electron-peer-reviewed-fulltext-article-COPD. 10.2147/COPD.S181224 PMC6305135 30587961

[17] Come migliorare la qualità di vita dei pazienti colpiti da bronco-pneumopatia cronica e dei loro caregiver? | Azienda Ospedaliera Nazionale SS. Antonio e Biagio e Cesare Arrigo Alessandria. [cited 2023 Nov 15]. Available from: https://www.ospedale.al.it/it/comunicazione/notizie/come-migliorare-qualita-vita-pazienti-colpiti-bronco-pneumopatia-cronica-loro-caregiver.

[18] Aiello F, Alunni A, Berardi M, Bordoni F, Calzolari M, Coviello AP, et al. Medicina Pratica L’associazione LABA-LAMA nella gestione del paziente con BPCO-Il punto di vista della Medicina Generale. Rivista Società Italiana di Medicina Generale n 3 • [Internet]. [cited 2023 Nov 15];29:2022. Available from: https://goldcopd.org/.

[19] ALLEGATOA alla Dgr n. 206 del 24 febbraio 2015.

[20] Miravitlles M, Soler-Cataluña JJ, Calle M, Molina J, Almagro P, Quintano JA, et al. Spanish Guidelines for Management of Chronic Obstructive Pulmonary Disease (GesEPOC) 2017. Pharmacological Treatment of Stable Phase. Arch Bronconeumol. 2017 Jun 1 [cited 2023 Nov 15];53(6):324–35. Available from: https://pubmed.ncbi.nlm.nih.gov/28477954/. 10.1016/j.arbres.2017.03.018 28477954

[21] NHS England » Ambitions for Palliative and End of Life Care: A national framework for local action 2021–2026. [cited 2023 May 29]. Available from: https://www.england.nhs.uk/publication/ambitions-for-palliative-and-end-of-life-care-a-national-framework-for-local-action-2021-2026/.

[22] POCKET GUIDE TO COPD DIAGNOSIS, MANAGEMENT, AND PREVENTION A Guide for Health Care Professionals. 2020 [cited 2023 May 29]; Available from: www.goldcopd.org.

[23] Rayner J, Khan T, Chan C, Wu C. Illustrating the patient journey through the care continuum: leveraging structured primary care electronic medical record (EMR) data in Ontario, Canada using chronic obstructive pulmonary disease as a case study. Int J Med Inform. 2020 Aug 1 [cited 2023 Jun 3];140. Available from: https://pubmed.ncbi.nlm.nih.gov/32473567/. 10.1016/j.ijmedinf.2020.104159 32473567

[24] Walker S, Andrew S, Hodson M, Roberts CM. Stage 1 development of a patient-reported experience measure (PREM) for chronic obstructive pulmonary disease (COPD). NPJ Prim Care Respir Med. 2017 Dec 1 [cited 2023 Jun 3];27(1). Available from: https://pubmed.ncbi.nlm.nih.gov/28740181/. 10.1038/s41533-017-0047-5 PMC5524786 28740181

[25] Agustí A, Celli BR, Criner GJ, Halpin D, Anzueto A, Barnes P, et al. Global initiative for chronic obstructive lung disease 2023 report: GOLD executive summary. Eur Respir J. 2023 Apr 1 [cited 2023 May 29];61(4):2300239. Available from: https://erj.ersjournals.com/content/61/4/2300239. 10.1183/13993003.00239-2023 PMC10066569 36858443

[26] Reddel HK, Bacharier LB, Bateman ED, Brightling CE, Brusselle GG, Buhl R, et al. Global Initiative for Asthma Strategy 2021 Executive Summary and Rationale for Key Changes. Am J Respir Crit Care Med [Internet]. 2022 Jan 1 [cited 2023 Jun 3];205(1):17–35. Available from: https://pubmed.ncbi.nlm.nih.gov/34658302/. 10.1164/rccm.202109-2205PP PMC8865583 34658302

[27] Jones PW, Watz H, Wouters EFM, Cazzola M. COPD: the patient perspective. Int J Chron Obstruct Pulmon Dis. 2016 [cited 2023 Jun 3];11 Spec Iss(Spec Iss):13–20. Available from: https://pubmed.ncbi.nlm.nih.gov/26937186/. 10.2147/COPD.S85977 PMC4764028 26937186

[28] Aggarwal AN, Gupta D, Jindal SK. The relationship between FEV1 and peak expiratory flow in patients with airways obstruction is poor. Chest. 2006 [cited 2023 Jun 3];130(5):1454–61. Available from: https://pubmed.ncbi.nlm.nih.gov/17099024/. 10.1378/chest.130.5.1454 17099024

[29] 2023 GOLD guidelines for chronic obstructive pulmonary disease. [cited 2023 Jun 26]. Available from: https://www.jwatch.org/na56004/2023/04/20/2023-gold-guidelines-chronic-obstructive-pulmonary-disease.

[30] Oliver SM. Living with failing lungs: the doctor–patient relationship. Fam Pract. 2001 Aug 1 [cited 2023 Jun 3];18(4):430–9. Available from: https://academic.oup.com/fampra/article/18/4/430/620192. 10.1093/fampra/18.4.430 11477052

[31] JIACI · J Invest Allergol Clin Immunol. [cited 2023 Jun 3]. Available from: https://www.jiaci.org/summary/vol16-issue4-num90.

[32] Martínez-Guiu J, Arroyo-Fernández I, Rubio R. Impact of patients’ attitudes and dynamics in needs and life experiences during their journey in COPD: an ethnographic study. Expert Rev Respir Med. 2022 [cited 2023 Jun 3];16(1):121–32. Available from: https://pubmed.ncbi.nlm.nih.gov/34238094/. 10.1080/17476348.2021.1891884 34238094

[33] Lainscak M, Gosker HR, Schols AMWJ. Chronic obstructive pulmonary disease patient journey: hospitalizations as window of opportunity for extra-pulmonary intervention. Curr Opin Clin Nutr Metab Care. 2013 May [cited 2023 Jun 3];16(3):278–83. Available from: https://pubmed.ncbi.nlm.nih.gov/23507875/. 10.1097/MCO.0b013e328360285d 23507875

[34] Kim-Dorner SJ, Schmidt T, Kuhlmann A, Graf von der Schulenburg JM, Welte T, Lingner H. Age- and gender-based comorbidity categories in general practitioner and pulmonology patients with COPD. NPJ Prim Care Respir Med. 2022 Dec 1 [cited 2023 Nov 15];32(1). Available from: /pmc/articles/PMC9061861/. 10.1038/s41533-022-00278-8 PMC9061861 35501357

[35] Maestri R, Vitacca M, Paneroni M, Zampogna E, Ambrosino N. Gender and age as determinants of success of pulmonary rehabilitation in individuals with chronic obstructive pulmonary disease. Arch Bronconeumol. 2023 Mar 1 [cited 2023 Nov 15];59(3):174–7. Available from: https://www.archbronconeumol.org/en-gender-age-as-determinants-success-articulo-S0300289622005683. 10.1016/j.arbres.2022.09.008 36192251

